# Dengue Virus Type 1 Infection in Traveler Returning from Benin to France, 2019

**DOI:** 10.3201/eid2608.200055

**Published:** 2020-08

**Authors:** Toscane Fourié, Léa Luciani, Sophie Amrane, Christine Zandotti, Isabelle Leparc-Goffart, Laetitia Ninove, Antoine Nougairède

**Affiliations:** Aix Marseille Université, Marseille, France (T. Fourié, L. Luciani, S. Amrane, C. Zandotti, I. Leparc-Goffart, L. Ninove, A. Nougairède);; French Armed Forces Biomedical Research Institute, Marseille (T. Fourié, I. Leparc-Goffart)

**Keywords:** dengue virus, viruses, infection, Benin, France, West Africa, traveler, vector-borne infections, mosquitoes, arboviruses

## Abstract

We investigated a case of dengue virus type 1 infection acquired in Benin. Phylogenetic analysis revealed the strain belongs to genotype V but clusters with Asian, rather than with known African, strains. Our finding suggests the introduction of Asian dengue virus in West Africa.

Dengue fever, a major public health concern throughout tropical and subtropical regions of the world, is a mosquitoborne disease caused by 4 distinct dengue virus (DENV) serotypes that share antigenic relationships (DENV-1–4). Although DENV is endemic to most countries in Africa, laboratory-confirmed dengue remains rare. Active transmission of DENV on the African continent lacks consistent molecular detection and characterization. In Benin, West Africa, probable cases of dengue fever have been described as far back as 1987 (*1*). In 2010, the first confirmed cases of dengue fever acquired in Benin were reported (*2*), and DENV-3 was identified (*3*). Since 2010, only a few cases have been reported in Benin (*4*). We investigated confirmed DENV-1 infection acquired in Benin in a traveler returning to France.

In February 2019, acute fever associated with intermittent headaches developed in a 16-year-old girl after she returned to her home in Marseille from a 10-day stay in urban Benin. She sought medical care 2 days after symptom onset and was hospitalized for suspected malaria at the public hospital of Marseille. She did not take antimalarial prophylaxis and did not experience symptoms during her stay in Benin. Physical examination revealed rash on her face, torso, and limbs.

At admission, laboratory results indicated a mild increase of aspartate transaminase (74 IU/L [reference <25 IU/L]), a moderate increase in C-reactive protein (25 mg/L [reference <5 mg/L]), and hypokalemia (potassium 3.07 mmol/L [reference 3.5–4.5 mmol/L]). The rapid diagnostic test result for malaria was negative. We detected DENV nonstructural protein 1 antigen (SD Bioline Dengue Duo Combo kit; Alere [Abbott], https://www.alere.com) and DENV RNA (*5*) in the patient’s serum collected at admission. Serotype-specific real-time reverse transcription PCR (*5*) identified DENV-1. DENV serology (ELISA; Euroimmun, https://www.euroimmun.com) showed absence of IgM and presence of IgG against DENV, suggesting the patient had been previously infected by other DENVs or serologically close flaviviruses. After 48 hours of hospitalization and symptomatic treatment (acetaminophen 2 g/d), the patient recovered without complications and was discharged. She and her mother (legal guardian) provided written consent to publish her clinical and biological data collected by the Assistance Publique Hôpitaux de Marseille during her stay.

We isolated DENV from a positive sample on C6/36 (American Type Culture Collection CRL-1660) cells. We obtained the complete viral genome sequence of this DENV-1 strain, named 2019/BJ/9943 (deposited in GenBank under accession no. MN600714), from cell culture supernatant using next-generation sequencing as previously described (*6*).

Phylogenetic analysis showed that the 2019/BJ/9943 strain belongs to DENV-1 genotype V (Figure). Within genotype V, American-Caribbean, Asian, and African clades are strongly associated with DENV strains detected in these specific geographic regions, suggesting a local dispersion of those viruses. The African clade of DENV-1 genotype V comprises only 3 full-genome sequences, all of which come from West Africa. Surprisingly, the 2019/BJ/9943 strain belongs to the Asian clade. The Asian clade already contains another strain from Africa isolated from the Comoros (an archipelago located between Madagascar and Mozambique) in 1993. Furthermore, a recent phylogenetic study in Nigeria based on envelop protein coding sequences suggests that certain DENV-1 genotype I strains from Nigeria cluster with strains from Cambodia (*7*). However, such partial sequences do not provide enough single-nucleotide polymorphisms for subgenotyping. In our study, full-genome phylogenetic analysis provides solid evidence that 2019/BJ/9943 strain is related to Asian strains.

**Figure:**
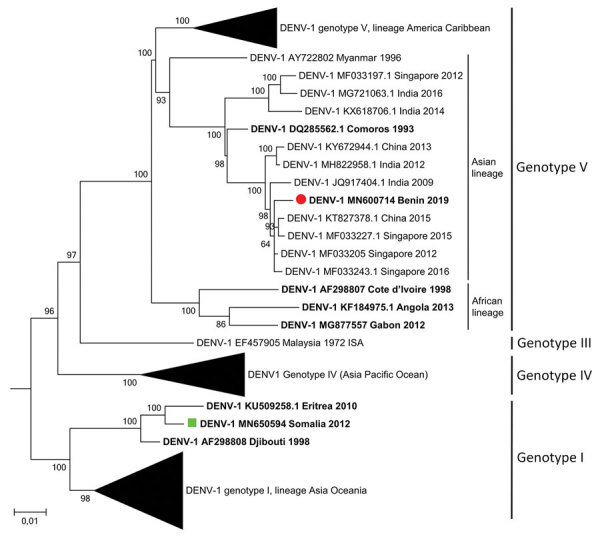
Maximum-likelihood phylogenetic tree of DENV-1 detected in a traveler who returned from Benin to France (red circle) along with other strains from Africa (bold), strain provided by the French National Reference Centre for Arbovirus (green square), and reference strains. The general time-reversible model (discrete γ distribution with evolutionarily Invariant sites) was used to construct the tree with 130 full-genome sequences. Bootstrap support values (percentage of 500 replicates) are shown at nodes. The tree was rooted with reference strains of DENV-2, DENV-3, and DENV-4 (not shown). Scale bar indicates nucleotide substitutions per site. DENV, dengue virus.

Altogether, these data suggest repeated introductions of DENV-1 strains from Asia to Africa. Considering the lack of molecular data on DENV-1 strains from West Africa, it is difficult to estimate when the 2019/BJ/9943 strain was introduced to the continent from Asia. However, after the 2008 financial crisis, an increased number of migrant workers from China arrived in West Africa because of a rapid development of economic exchange and a gold rush in Ghana in 2013 (*8*). Additional molecular data from West Africa are needed to determine the effect of these population flows on the circulation of DENV between Asia and West Africa and to further clarify the epidemiology for this virus of serious public health concern.

This work was supported by the European Virus Archive Goes Global Project (European Union—Horizon 2020 program under grant agreement no. 653316; http://www.european-virus-archive.com) and the French Armed Forces Biomedical Research Institute.
